# Hypoxic HPV‐Positive Cancer Cells Are Particularly Sensitive to the Pro‐Senescent Effects of B‐MYB Repression Due to the Lack of Compensatory A‐MYB Induction

**DOI:** 10.1002/jmv.70422

**Published:** 2025-05-30

**Authors:** Milica Velimirović, Alicia Avenhaus, Claudia Lohrey, Julia Bulkescher, Felix Hoppe‐Seyler, Karin Hoppe‐Seyler

**Affiliations:** ^1^ German Cancer Research Center (DKFZ), Molecular Therapy of Virus‐Associated Cancers Heidelberg Germany; ^2^ Faculty of Biosciences Heidelberg University Heidelberg Germany

**Keywords:** A‐MYB, B‐MYB, cervical cancer, human papillomavirus (HPV), hypoxia, senescence

## Abstract

Tumor hypoxia is typically linked to increased therapy resistance and poor prognosis of many malignancies, including HPV‐positive cancers. One possible resistance mechanism is the increased resistance of hypoxic tumor cells to cellular senescence. It is thus highly interesting to identify strategies which could increase their pro‐senescent susceptibility. In comparative analyses of normoxic and hypoxic HPV‐positive cancer cells, we here uncover that the interconnection between B‐MYB and its paralog A‐MYB plays a key role for their senescence response, but shows a differential regulation under normoxia and hypoxia. In specific, we demonstrate that the pro‐senescent response to B‐MYB loss is counteracted by a compensatory upregulation of A‐MYB under normoxia. Therefore, efficient induction of senescence in normoxic cells requires the downregulation of both B‐MYB and A‐MYB. Interestingly, this compensatory A‐MYB induction is absent under hypoxia, rendering hypoxic cancer cells particularly sensitive to the pro‐senescent effect of B‐MYB repression. We further show that these regulatory effects are not confined to HPV‐positive cancer cells, indicating that they could be broadly conserved between different cancer types. Collectively, our findings reveal that hypoxic cancer cells are particularly sensitive to B‐MYB inhibition, which could provide a new strategy to target this therapeutically challenging cancer cell population.

## 1 Introduction

Oncogenic types of human papillomaviruses (HPVs), such as HPV16 or HPV18, are closely linked to the development of prevalent human cancers, including almost all cases of cervical cancer and a substantial portion of oropharyngeal cancers [1, 2, 3]. The sustained expression of the viral *E6/E7* oncogenes is essential for maintaining the proliferation of HPV‐positive cancer cells [1, 4, 5]. Interfering with E6/E7 expression has been shown to result in a rapid and efficient induction of cellular senescence [6, 7], which is classically defined as an irreversible growth arrest [8]. Thus, interfering with the expression or function of the HPV *E6/E7* oncogenes could possibly serve as a pro‐senescent therapeutic strategy for the treatment of HPV‐positive cancers [9, 10].

This view, however, is complicated by the behavior of HPV‐positive cancer cells under hypoxic conditions, as they are often present in subregions of solid tumors, including cervical and oropharyngeal cancers. Importantly, the degree of hypoxia in cancers typically correlates with increased therapy resistance and poor patient prognosis in the clinic [11], which is also observed for HPV‐positive cancers [12]. This cellular phenotype could be supported by the increased resistance of hypoxic tumor cells to the induction of cellular senescence [13, 14], thereby interfering with an important tumor suppressor pathway [8]. We previously found that the viral *E6/E7* oncogene expression is strongly downregulated in hypoxic HPV‐positive cancer cells. Yet, other than normoxic HPV‐positive cancer cells, they do not senesce, despite E6/E7 repression, and they are only reversibly growth arrested, resuming proliferation upon reoxygenation [15, 16]. These observations indicate that hypoxic HPV‐positive cancer cells possess an increased resistance towards therapeutic strategies targeting the viral oncogenes. Moreover, the ability of hypoxic HPV‐positive cancer cells to evade senescence and resume growth upon reoxygenation may contribute to tumor recurrence following treatment when surviving cancer cells regain access to increased oxygen supply, e.g. as result of therapy‐induced tumor shrinkage [17] or neovascularization [18]. Thus, it is of high importance to gain insights into the mechanisms by which hypoxic HPV‐positive cancer cells avoid senescence and to investigate whether it may be possible to increase their senescence susceptibility.

The B‐MYB oncoprotein is widely recognized as a key cellular transcription factor which can affect crucial proliferation‐linked processes, including cell cycle, apoptosis, and senescence control, by undergoing intricate interactions with multiple signaling pathways [19, 20]. In this study, we show that B‐MYB and its paralog A‐MYB are critical determinants for the differential senescence response of normoxic and hypoxic HPV‐positive cancer cells. We further reveal that hypoxic cells are particularly vulnerable to the pro‐senescent effects of B‐MYB silencing, since they are unable to functionally compensate for B‐MYB loss through induction of A‐MYB expression. Besides uncovering a crucial role for the B‐MYB/A‐MYB interconnection in the control of cellular senescence, these findings also possess strategic implications for therapeutically targeting hypoxic cancer cells.

## 2 Materials and Methods

### 2.1 Cell Culture and Treatment Conditions

HPV18‐positive HeLa (RRID:CVCL_0030), HPV16‐positive SiHa (RRID:CVCL_0032) and CaSki (RRID:CVCL_1100) cervical cancer cells, as well as HPV‐negative U2OS (RRID:CVCL_0042) osteosarcoma and HCT116 (RRID:CVCL_0291) colon cancer cells were obtained from the tumor bank of the German Cancer Research Center (DKFZ), Heidelberg. HeLa‐mKate2 and SiHa‐mKate2 cells were generated as previously described [21]. Cell lines were certified negative for mycoplasma contamination by PCR, and authentication was performed by single‐nucleotide polymorphism profiling within the last year (Multiplexion, GmbH). Cells were cultured at 37°C under normoxia (21% O_2_, 5% CO_2_), or hypoxia (1% O_2_, 5% CO_2_) in Dulbecco's minimal essential medium (DMEM) containing 1 g/L glucose (HeLa, SiHa, CaSki, U2OS), or McCoy's 5 A medium (HCT116) (Gibco, Thermo Fisher Scientific) and supplemented with 10% fetal bovine serum (PAN‐Biotech), 2 mM L‐glutamine, 100 U/mL penicillin, and 100 μg/mL streptomycin (Sigma‐Aldrich). If indicated, nocodazole (Cayman Chemical Company) dissolved in dimethyl sulfoxide (DMSO) was added into the cell culture medium at a final concentration of 0.1 µg/mL. DMSO served as solvent control. Experiments under hypoxic conditions were performed in a hypoxic chamber (InvivO_2_ 400 physiological oxygen workstation, Ruskinn Technology Ltd.).

### 2.2 Small Interfering RNA (siRNA) Transfections

Silencer Select siRNAs were chemically synthesized (Life Technologies, Thermo Fisher Scientific) and transfected with Lipofectamine RNAiMAX (Invitrogen, Thermo Fisher Scientific) either individually or as an equimolar pool at a final siRNA concentration of 2–10 nM according to the manufacturer's protocol. For cotransfection experiments total siRNA concentration was kept constant across all samples by supplementing with control siRNA (siNeg).

The siRNA target sequences were: siB‐MYB‐1: 5′‐CUGGAACUCUACCAUCAAA‐3′, siB‐MYB‐2: 5′‐GAAACAUGCUGCGUUUGUA‐3′, siB‐MYB‐3: 5′‐GAUCUGGAUGAGCUGCACU‐3′, siA‐MYB‐1: 5′‐GGAAGAAGAUAUUCGGGAA‐3′, siA‐MYB‐2: 5′‐CGAGCACACUAGUGAGUUU‐3′, and control siRNAs, siContr‐1: 5′‐CAGUCGCGUUUGCGACUGG‐3′ or siNeg: 5′‐UACGACCGGUCUAUCGUAG‐3′, both of which contain at least four mismatches to all known human genes. Equimolar pools of three different HPV18 and HPV16 E6 or E6/E7‐targeting siRNAs (siE6 and siE6/E7) have been characterized previously [15].

### 2.3 RNA Preparation and Quantitative Reverse Transcription‐Polymerase Chain Reaction (qRT‐PCR)

RNA extraction and qRT‐PCR were performed as described previously [22]. Primer sequences were as follows: CCNA2 for: 5′‐CCCCCAGAAGTAGCAGAGTTT‐3′; CCNA2 rev: 5′‐ACTTGAGGTATGGGTCAGCATC‐3′; CCNB1 for: 5′‐GCCTCTACCTTTGCACTTCCT‐3′; CCNB1 rev: 5′‐TGTTGTAGAGTTGGTGTCCATT‐3′; CCNB2 for: 5′‐AAGTTCCAGTTCAACCCACCA‐3′; CCNB2 rev: 5′‐CCTCAGGTGTGGGAGAAGGA‐3′; CDC25C for: 5′‐TGGAAACTTGGTGGACAGTGA‐3′; CDC25C rev: 5′‐GGGAGCGATATAGGCCACTT‐3′; MYBL1 for: 5′‐GCGAACTTAGGGATGGCTCA‐3′; MYBL1 rev: 5′‐TGCCCACAAATAGGGGTTGA‐3′; MYBL2 for: 5′‐AGGCTGGCATCGAACTCATC‐3′; MYBL2 rev: 5′‐CTTGGGCAGTGTGGACATCA‐3′; PLK1 for: 5′‐TGTTAGTGGGCAAACCACCTT‐3′; PLK1 rev: 5′‐GGCAGTGGGATCTGTCTGAA‐3′; TMBIM6 for: 5′‐GTGGTCATGTGTGGCTTCGT‐3′; TMBIM6 rev: 5′‐GGAAAGGCTGGATGGTCACT‐3′. All qRT‐PCR analyses were performed in duplicates from at least three independent biological replicates and relative mRNA levels were quantified using the comparative Ct (2^−ΔΔCt^) method [23]. Ct values were normalized against *TMBIM6*, used as an internal reference gene suitable for assessing hypoxia‐related gene expression in cervical cancer samples [24]. Fold change values were log_2_ transformed and subsequently analysed.

### 2.4 Immunoblot Analyses

SDS‐polyacrylamide gel electrophoresis (SDS‐PAGE) and Western blot analyses were performed as detailed previously [22]. Following primary antibodies were used: anti‐HPV18 E6 (AVC 399) and anti‐HPV16 E6 (AVC 843, kind gifts from Dr. Johannes Schweizer, Arbor Vita Corporation, Fremont, CA, USA); anti‐HPV18 E7 (E7C) [25]; anti‐HPV16 E7 (NM2, kind gift from Dr. Martin Müller, German Cancer Research Center, Heidelberg, Germany); anti‐B‐MYB [26] (LX015.1, from Dr. Roger Watson; kindly provided by Dr. Stefan Gaubatz, University of Würzburg, Würzburg, Germany); anti‐β‐Actin (C4, sc‐47778), anti‐CDC25C (H‐6, sc‐13138), anti‐Cyclin A (H‐432, sc‐751), anti‐Cyclin B2 (A‐2, sc‐28303), anti‐Cyclin D1 (DCS‐6, sc‐20044), anti‐E2F‐4 (D‐7, sc‐398543), anti‐glyceraldehyde 3‐phosphate dehydrogenase (GAPDH) (FL‐335, sc‐25778), anti‐HIF‐2α (sc‐46691), anti‐PLK1 (F‐8, sc‐17783), anti‐Vinculin (7F9, sc‐73614), all from Santa Cruz Biotechnology; anti‐cleaved Caspase 9 (#7237), anti‐cleaved poly(ADP‐ribose) polymerase (PARP) (#9546), anti‐RBL2 (#13610), all from Cell Signaling Technology; anti‐Cyclin B1 (05‐373, clone GNS3 [8A5D12]), from Upstate; anti‐A‐MYB (HPA008791) from Sigma‐Aldrich; anti‐HIF‐1α (610959) from BD Biosciences. The following secondary antibodies were used: anti‐mouse IgG (H + L), HRP conjugate (W4021) and anti‐rabbit IgG (H + L), HRP conjugate (W4011) (Promega), and anti‐chicken IgY‐HRP (sc‐2428) (Santa Cruz Biotechnology). Enhanced luminescence (ECL) reagents WesternBright Sirius (Advansta) or Amersham ECL Prime (Cytiva) were used to visualize immunoblots. All immunoblots were repeated at least three times, with consistent results.

### 2.5 Flow Cytometry and EdU Assay

For cell cycle analyses, cells were trypsinized, washed in cold phosphate‐buffered saline (PBS), and fixed with 75% ice‐cold ethanol at −20°C overnight. Cells were pelleted and DNA was stained with propidium iodide (Sigma‐Aldrich) at a final concentration of 25 µg/mL in the presence of RNase A (500 µg/mL, Roche Diagnostics). DNA content per cell was measured using the BD LSRFortessaTM (BD Biosciences) and the BD FACS DIVA Software version v8.0.1. 10 000–20 000 cells were counted per condition in every experiment. Data analysis was carried out using FlowJoTM v10.8.1 Software (BD Life Sciences). Quantification of individual cell cycle phases was based on the Dean‐Jett‐Fox model [27]. Cell cycle analyses were conducted three times, with consistent results. To measure DNA synthesis, a flow cytometry‐based EdU assay using EdU‐Click 647 Kit (baseclick) was employed according to the manufacturer's protocol. Analysis was carried out as detailed for cell cycle analyses.

### 2.6 Senescence and Colony Formation Assays

Following treatment as outlined in the text, cells were split, re‐seeded in fresh medium and cultivated under standard cell culture conditions (21% O_2_, 5% CO_2_, 37°C). For senescence assays, cells were fixed with 2% formaldehyde and 0.2% glutaraldehyde in PBS. Fixed cells were then stained for Senescence‐Associated β‐Galactosidase (SA‐β‐Gal) activity (1 mg/mL X‐Gal, 5 mM potassium ferricyanide, 5 mM potassium ferrocyanide, 40 mM citric acid, 150 mM NaCl, and 2 mM MgCl2 at pH 6.0) at 37°C for 16–24 h. Imaging of cells was performed on the EVOSxl Core Cell Imaging System (Invitrogen, Thermo Fisher Scientific) using a 20‐fold magnification. For colony formation assays (CFAs), colonies were fixed with formaldehyde and stained with crystal violet. All senescence and colony formation assay experiments were repeated at least three times, with consistent results.

### 2.7 Live‐Cell Imaging

Live‐cell imaging was conducted with the Incucyte S3 device (Sartorius) to monitor mKate2‐labeled cells. Cells were reverse transfected and seeded in 96‐well plates at a density of 2000 (HeLa‐mKate2) or 3000 (SiHa‐mKate2) cells per well. Images of each well were captured from 24 h post‐transfection every 4 or every 6 h, using a 10‐fold magnification. Cell proliferation was assessed by counting viable cells (red‐labeled objects) for up to 96 h using the Incucyte 2021C software. Red object counts are shown relative to the counts at 24 h post‐transfection (set as 1).

### 2.8 Statistical Analysis

GraphPad Prism 10.1.2 (GraphPad Software Inc.) was used for statistical tests. A two‐sided unpaired *t*‐test, one‐way analysis of variance (ANOVA), followed by Sidak's or Dunnett's multiple comparisons test, or two‐way ANOVA, followed by Fisher's LSD test, were conducted to determine statistical significance, indicated as *p ≤ 0.05, **p ≤ 0.01, and ***p ≤ 0.001.

## 3 Results

### 3.1 Cell Cycle Regulators Are Differentially Regulated in HPV‐Positive Cancer Cells Upon E6/E7 Repression Under Normoxia or by Hypoxia

Previous studies have shown that RNAi (RNA interference)‐mediated E6/E7 repression under normoxia as well as the hypoxia‐associated E6/E7 repression are both linked to efficient inhibition of the proliferation of HPV‐positive cancer cells. Notably, however, this proliferative stop becomes irreversible through induction of senescence when E6/E7 is blocked by RNAi under normoxia, whereas it remains reversible when E6/E7 expression is repressed by hypoxia [15, 16, 28]. To gain insights into the mechanisms underlying these discrepant growth responses, the response of cell cycle regulatory factors in HPV‐positive cancer cells was comparatively analyzed under normoxia (N; 21% O_2_) and hypoxia (H; 1% O_2_).

**Figure 1:**
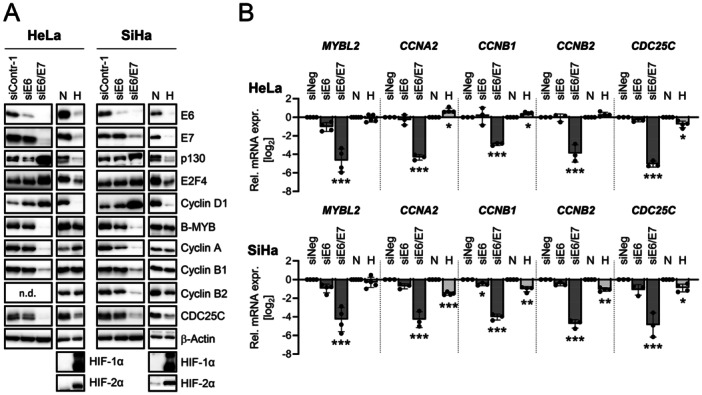
E6/E7 repression under normoxia or by hypoxia: differential expression of cell cycle regulators. HeLa or SiHa cells were transfected with siE6, siE6/E7, or control siRNA (siContr‐1 or siNeg) and grown for 72 h (left panels for each cell line) or were cultivated under normoxia (N; 21% O_2_) or hypoxia (H; 1% O_2_) for 24 h (right panels for each cell line). (A) Immunoblot analyses of E6, E7, p130, E2F4, Cyclin D1, B‐MYB, Cyclin A, Cyclin B1, Cyclin B2, CDC25C, hypoxia markers HIF‐1α and HIF‐2α, and β‐Actin protein levels; n.d., not determined. (B) qRT‐PCR analyses of *MYBL2* (coding for B‐MYB), *CCNA2* (coding for Cyclin A2), *CCNB1* (coding for Cyclin B1), *CCNB2* (coding for Cyclin B2), and *CDC25C* mRNA levels. Individual data points and mean expression levels with standard deviation (SD) (*n* ≥ 3) relative to the expression of siNeg or at normoxia, respectively, are shown (log_2_). Statistical significance was evaluated using one‐way ANOVA (siNeg, siE6, siE6/E7) or a two‐sided *t*‐test (N, H). *p ≤ 0.05, **p ≤ 0.01, and ***p ≤ 0.001.

Normoxic HPV18‐positive HeLa and HPV16‐positive SiHa cells were transfected with siRNA pools blocking either *E6* oncogene expression alone or *E6/E7* oncogene expression in combination (Figure 1). Notably, unlike E6 repression alone, combined E6/E7 repression, which rapidly induces highly efficient cellular senescence in normoxic HPV‐positive cancer cells [6, 7, 15, 28], was linked to a strong reduction in the levels of proteins that promote cell cycle progression, including B‐MYB, Cyclin A, Cyclin B1, Cyclin B2, and CDC25C (Figure 1A, left panels for each cell line). qRT‐PCR analyses revealed that their downregulation occurs at the transcript level (Figure 1B). In contrast to the downregulation of these cell cycle‐promoting factors, the expression of proteins inhibiting cell cycle progression, such as the DREAM (dimerization partner, RB‐like, E2F and multi‐vulval class B) complex components p130 and E2F4 [29, 30], was increased upon E6/E7 repression (Figure 1A). Further, Cyclin D1 levels, which are upregulated in senescent cells [31], were increased, in line with the strong pro‐senescent effects of E6/E7 repression under normoxia.

Remarkably, whereas hypoxia also led to a strong downregulation of HPV E6/E7 expression (Figure 1A, right panels for each cell line), as expected [15, 16], the response of cell cycle regulators strongly differed. Cell cycle‐promoting proteins, which were strongly downregulated by RNAi‐mediated E6/E7 repression under normoxia, namely B‐MYB, Cyclin A, Cyclin B1, Cyclin B2, and CDC25C, were either not or much less downregulated when E6/E7 repression occurred under hypoxia. These regulatory differences were also mirrored at the transcript level (Figure 1B). Moreover, whereas RNAi‐mediated E6/E7 repression under normoxia led to upregulation of p130, E2F4, and Cyclin D1 protein levels, they were downregulated in hypoxic cells despite E6/E7 repression (Figure 1A).

### 3.2 Hypoxia Blocks the Proliferation of HPV‐Positive Cancer Cells Without Substantially Affecting Their Cell Cycle Profile

**Figure 2:**
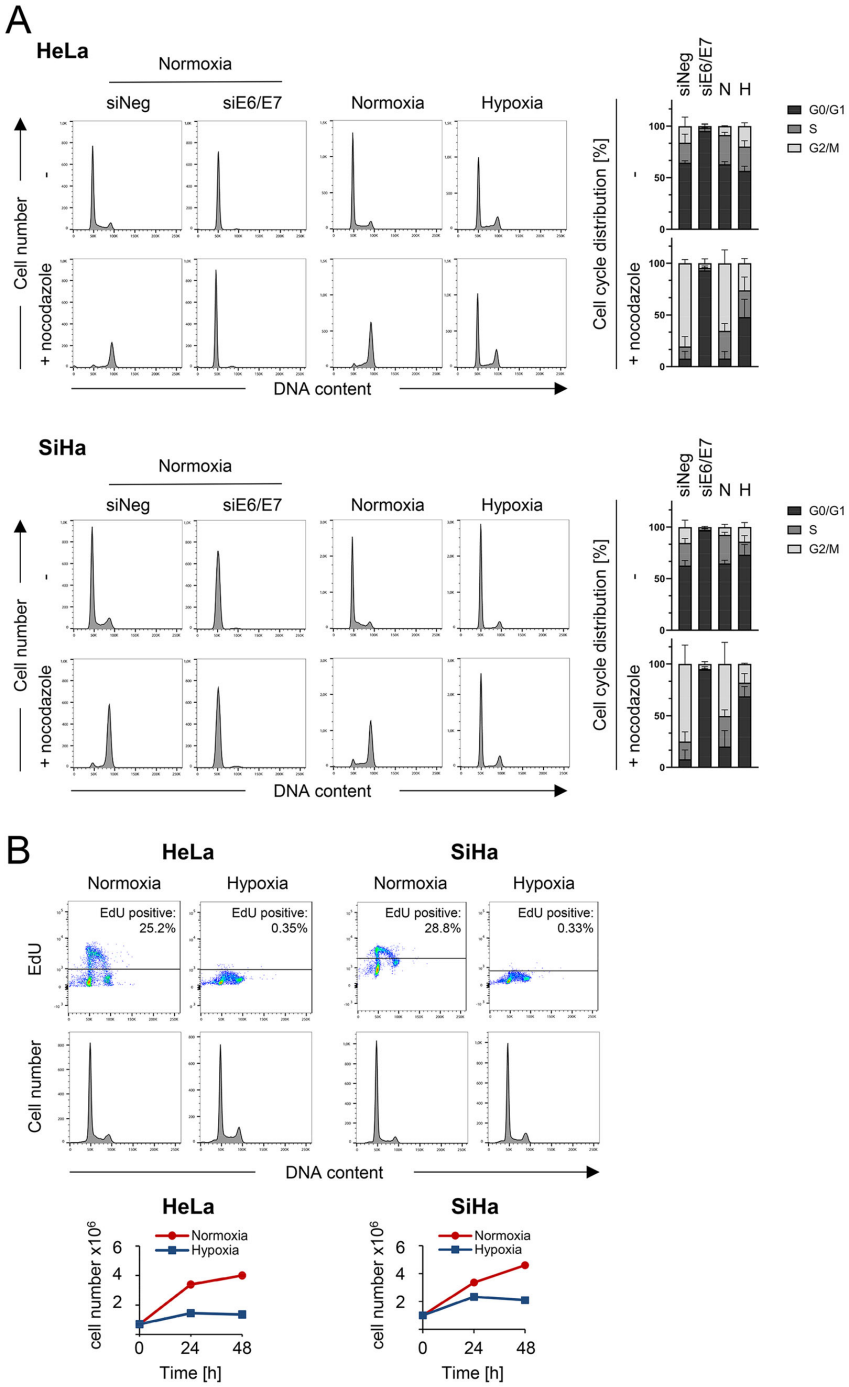
Hypoxia blocks the proliferation of HPV‐positive cancer cells without substantially affecting their cell cycle profiles. (A) HeLa or SiHa cells were transfected with siE6/E7 or control siRNA (siNeg) and grown for 72 h or were cultivated under normoxia or hypoxia for 48 h. Nocodazole or solvent control were added for the last 20 h of cultivation, as indicated. Cells were analyzed by flow cytometry and cell cycle profiles from representative experiments are shown (left panels). Averaged percentages of cell populations in the individual cell cycle phases from three different experiments with SD are indicated (right panels). (B) Upper panels: EdU incorporation and corresponding cell cycle analyses of HeLa and SiHa cells cultivated under normoxia or hypoxia for 24 h. Lower panels: Growth curves of HeLa or SiHa cells measured in parallel to the respective EdU assays. Viable cell numbers were determined by a trypan blue exclusion test of cells cultivated under normoxia or hypoxia for the indicated time periods.

In view of these discrepant responses of cell cycle regulators, the cell cycle profiles of HPV‐positive cancer cells under normoxia and hypoxia were comparatively analyzed by flow cytometry. Notably, whereas RNAi‐mediated E6/E7 repression in normoxic HeLa and SiHa cells resulted in a highly efficient G1 arrest, hypoxic cells exhibited only a modest increase in G1 and/or G2 populations, despite E6/E7 downregulation (Figure 2A, upper panels for each cell line). Cells were further treated with the microtubule inhibitor nocodazole, which targets proliferating cells by inducing a G2/M cell cycle arrest [32]. Accordingly, proliferating, normoxic HPV‐positive cells (either control siRNA‐transfected or untransfected) that were treated with nocodazole exhibited a pronounced accumulation in the G2/M phase (Figure 2A, lower panels for each cell line).

In contrast, upon RNAi‐mediated E6/E7 repression in HPV‐positive cancer cells, the cell cycle profile of normoxic cells did not appreciably change under nocodazole treatment (Figure 2A, lower panels for each cell line), showing that these cells are efficiently growth inhibited. Of note, albeit hypoxic HeLa and SiHa cells exhibited a cell cycle profile which resembles that of proliferating, normoxic cells, their cell cycle distribution was, if at all, only modestly affected by nocodazole treatment. This indicates that they are effectively growth arrested, despite exhibiting a largely unchanged cell cycle profile. Additionally, we performed EdU assays in HeLa and SiHa cells (Figure 2B), which in both cell lines revealed inhibition of DNA synthesis in the S‐phase under hypoxic conditions, indicating that DNA replication was halted. Accordingly, corresponding growth curves, accompanying the EdU assays, showed that proliferation was efficiently inhibited under hypoxia, consistent with previous results [15, 16].

Similar to the behavior of HPV‐positive cells, treatment of normoxic HPV‐negative U2OS osteosarcoma cells with nocodazole led to a strong increase in G2/M populations, which was much less pronounced under hypoxia (Supporting Information S1: Figure S1A). Further, the effects of hypoxia on the expression of cell cycle regulatory proteins in U2OS and HPV‐positive cancer cells were similar (Figure 1A and Supporting Information S1: Figure S1B). These findings indicate that the cell cycle response of HPV‐positive cancer cells to hypoxia is largely E6/E7‐independent.

Collectively, these results show that the anti‐proliferative effects of hypoxia in HPV‐positive cancer cells are not associated with a pronounced accumulation of cells in a distinct cell cycle phase, despite efficient E6/E7 repression. This strongly contrasts their response to RNAi‐mediated E6/E7 repression under normoxia, where growth inhibition is linked to a highly efficient G1 arrest and subsequent senescence induction.

### 3.3 The Interplay Between B‐MYB and A‐MYB Is a Key Factor for Determining the Proliferation Rate of HPV‐Positive and HPV‐Negative Cancer Cells

We observed that B‐MYB levels were strongly downregulated when HPV‐positive cancer cells induce senescence (RNAi‐mediated E6/E7 repression under normoxia), whereas they were maintained when cells evade senescence (hypoxia‐linked E6/E7 repression) (Figure 1A,B). This differential regulation raised our particular interest, since B‐MYB loss has been reported to promote senescence in normoxic cells [19, 20]. Moreover, a recent study has uncovered that the expression of B‐MYB paralog A‐MYB can be induced upon B‐MYB loss and has the potential to functionally substitute for B‐MYB by transcriptionally activating an overlapping spectrum of late cell cycle‐promoting genes [33]. We therefore next focused on analyzing the significance of B‐MYB and A‐MYB for the proliferation and senescence control of HPV‐positive cancer cells, starting with analyses of cells cultivated under normoxia.

**Figure 3:**
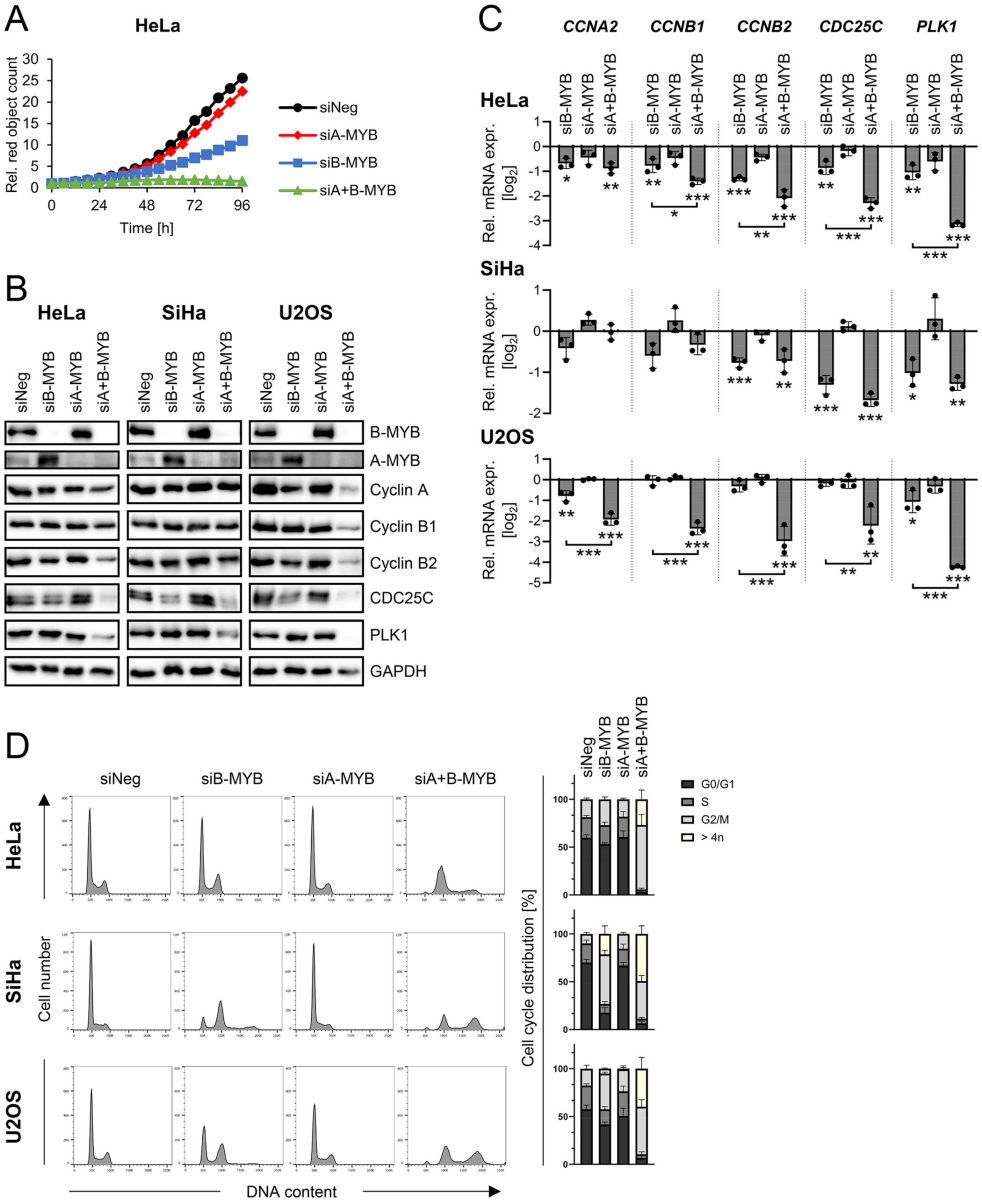
A‐MYB repression enhances the effects of B‐MYB silencing on the proliferation and cell cycle regulation in normoxic cells. (A) Live‐cell imaging (Incucyte S3 live‐cell imaging system) was used to determine cell counts of HeLa‐mKate2 cells transfected with siA‐MYB, siB‐MYB, either alone or in combination, or control siRNA (siNeg), and cultivated under normoxia for up to 96 h. (B) HeLa, SiHa, or U2OS cells were transfected with siA‐MYB, siB‐MYB, either alone or in combination, or control siRNA (siNeg) and harvested after 48 h (HeLa) or 72 h (SiHa, U2OS). Immunoblot analyses of B‐MYB, A‐MYB, Cyclin A, Cyclin B1, Cyclin B2, CDC25C, PLK1, and GAPDH protein levels. (C) Corresponding qRT‐PCR analyses of *CCNA2*, *CCNB1*, *CCNB2*, *CDC25C*, and *PLK1* mRNA levels. Individual data points and mean expression levels with SD (*n* = 3) relative to the expression of siNeg are shown (log_2_). Statistical significance was evaluated using one‐way ANOVA. *p ≤ 0.05, **p ≤ 0.01, and ***p ≤ 0.001. (D) Cells were analyzed by flow cytometry and cell cycle profiles from representative experiments are shown (left panels). Averaged percentages of cell populations in the individual cell cycle phases from 3 different experiments with SD are indicated (right panels).

In agreement with the documented growth‐promoting potential of B‐MYB in many cell types [19, 20], silencing of B‐MYB expression exerted anti‐proliferative effects in normoxic HeLa and SiHa cells (Figure 3A and Supporting Information S1: Figure S2). Interestingly, although A‐MYB silencing alone did not appreciably alter the proliferation rate, the anti‐proliferative effect of B‐MYB silencing was strongly enhanced when A‐MYB was silenced in parallel (Figure 3A; please refer to Supporting Information S1: Figure S2 for the validation of the employed siRNAs). These findings are compatible with the conception that the anti‐proliferative effects of B‐MYB loss in HPV‐positive cancer cells could be, at least partially, compensated by a reactive induction of A‐MYB and, furthermore, that a highly efficient growth inhibition requires repression of both B‐MYB and A‐MYB.

In support of this model, immunoblot analyses showed that both HeLa and SiHa cells exhibited a clear upregulation of A‐MYB levels following B‐MYB silencing (Figure 3B). Further, analyses of a selection of B‐MYB target genes that promote G2/M cell cycle progression revealed that the impact of B‐MYB loss differs to some degree across the examined cell lines, with most genes showing no significant changes in expression (Figure 3B and Supporting Information S1: Figure S3A,B). This response was not confined to HPV‐positive cancer cells, since it was also detectable in HPV‐negative U2OS (Figure 3B) and HCT116 colon cancer cells (Supporting Information S1: Figure S3A,B). Notably, however, combined silencing of both B‐MYB and A‐MYB expression led to a more pronounced downregulation of several target genes, with CDC25C and PLK1 expression being consistently strongly reduced across all analyzed cell lines, both on protein (Figure 3B and Supporting Information S1: Figure S3A) and RNA (Figure 3C and Supporting Information: Figure S3B) level.

Moreover, cell cycle analyses revealed that A‐MYB silencing alone had only weak effects, if at all, on the cell cycle profiles of HeLa, SiHa, CaSki, U2OS, and HCT116 cells (Figure 3D and Supporting Information S1: Figure S3C). In contrast, we observed an increase in G2/M populations following B‐MYB silencing, which was very pronounced in SiHa, readily detectable in CaSki, HCT116, and U2OS, and only subtle in HeLa cells. These findings are in line with reports showing that the cell cycle response to B‐MYB loss can exhibit cell type‐dependent variability [33, 34, 35]. Importantly, in all studied cell lines, the combined silencing of both B‐MYB and A‐MYB expression led to a much more efficient G2/M arrest compared to B‐MYB silencing alone and furthermore resulted in increased endoreduplication events ( > 4n), in line with a recent study [33] (Figure 3D and Supporting Information S1: Figure S3C).

Collectively, these findings indicate that the anti‐proliferative effects resulting from B‐MYB loss can be attenuated by a reactive increase of A‐MYB levels in both HPV‐positive and HPV‐negative cancer cells. Further, upon B‐MYB silencing, a concomitant interference with A‐MYB expression is essential to achieve a more profound downregulation of B‐MYB target genes, a strongly enhanced G2/M cell cycle arrest, and effective growth inhibition.

### 3.4 The Pro‐Senescent Effect of B‐MYB Downregulation in Normoxic Cells Is Enhanced by Concomitant A‐MYB Repression

**Figure 4:**
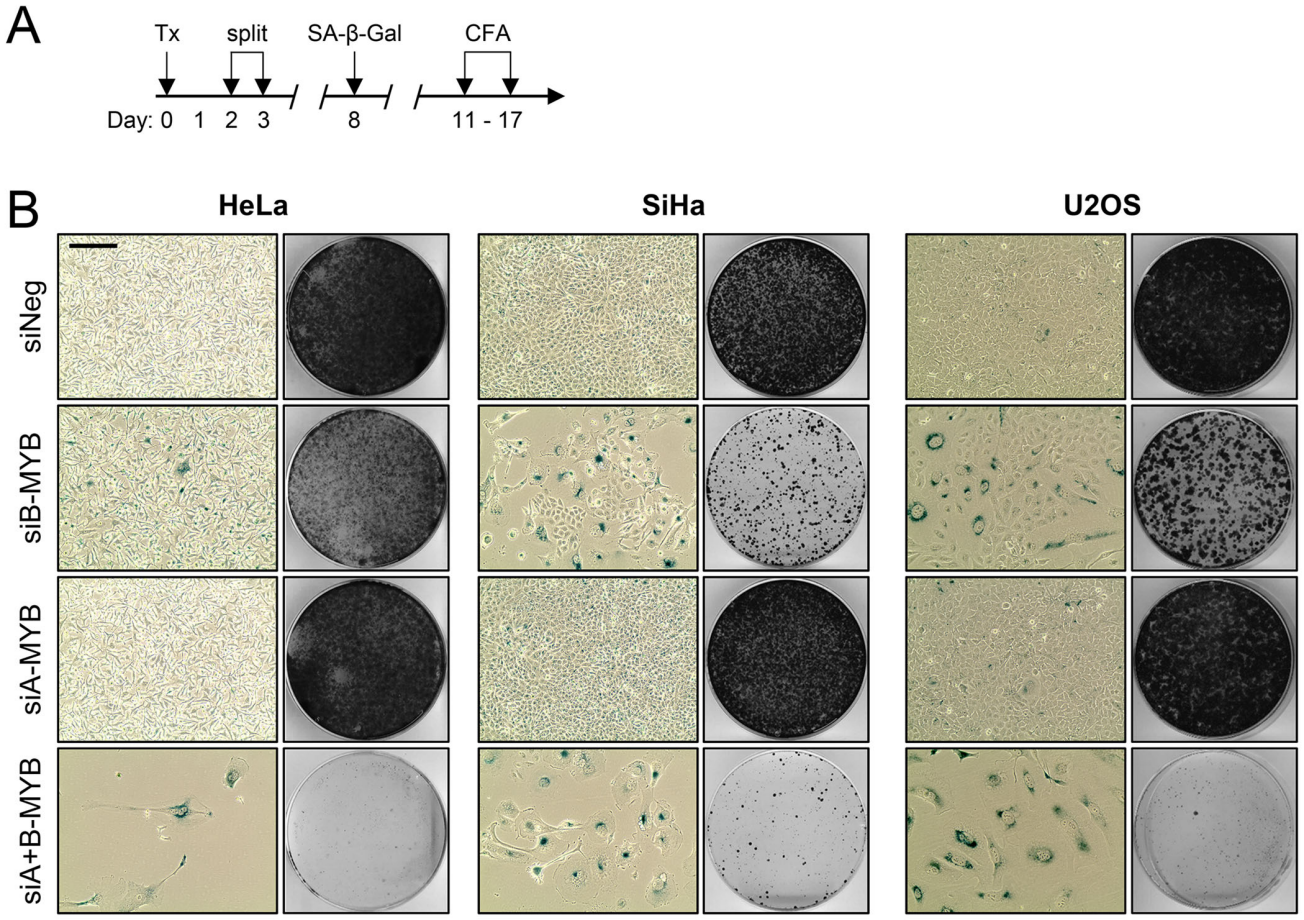
A‐MYB knockdown enhances the pro‐senescent effect of B‐MYB silencing in normoxic cells. (A) Treatment scheme: HeLa, SiHa, or U2OS cells were transfected (Tx) with siA‐MYB, siB‐MYB, either alone or in combination, or control siRNA (siNeg). Cells were split after 48 h (HeLa) or 72 h (SiHa, U2OS), and fixed after the indicated time periods for senescence assays (SA‐β‐Gal staining) or colony formation assays (CFAs). (B) SA‐β‐Gal assays (left panels; positive cells staining blue) (scale bar: 200 µm) and corresponding CFAs (right panels) of HeLa, SiHa, or U2OS cells.

To the best of our knowledge, whether the interconnection between B‐MYB and A‐MYB expression can affect the regulation of cellular senescence has not been investigated thus far. Following the treatment scheme depicted in Figure 4A, we found that A‐MYB silencing alone did not induce senescence in normoxic cells, whereas we observed readily detectable pro‐senescent effects upon B‐MYB silencing alone in SiHa, CaSki, U2OS, and HCT116 cells. This was shown by positive staining for the well‐established senescence marker SA‐β‐Gal (Senescence‐Associated‐β‐Galactosidase) and the typical morphological changes of senescent cells (flattening and enlargement of the cells) (Figure 4B and Supporting Information S1: Figure S3D, left panels for each cell line). In line, B‐MYB silencing was linked to a reduction in their colony formation capacities (Figure 4B and Supporting Information S1: Figure S3D, right panels for each cell line), as expected from the irreversible growth arrest associated with senescence. In normoxic HeLa cells, a pro‐senescent effect of B‐MYB silencing was only marginally detectable. Noteworthy, although B‐MYB silencing was highly efficient and resulted in virtually undetectable B‐MYB protein levels (Figure 3B and Supporting Information S1: Figure S3A), senescence induction was only partial in all investigated cell lines, as indicated by the emergence of cells lacking signs of senescence, next to senescent cells (Figure 4B and Supporting Information S1: Figure S3D, left panels for each cell line).

Importantly, the pro‐senescent response towards B‐MYB silencing was substantially enhanced in SiHa, CaSki, U2OS, and HCT116 cells by concomitant A‐MYB silencing, as shown by the strong reduction of cells that are capable to evade senescence (i.e. staining negative for SA‐β‐Gal and lacking the morphological signs of senescence) and increased impairment of colony formation capacity (Figure 4B and Supporting Information S1: Figure S3D). In HeLa cells, combined silencing of B‐MYB and A‐MYB caused a substantial decrease in cell numbers with a few residual cells being senescent. Since a recent study has reported pro‐apoptotic effects of combined B‐MYB and A‐MYB silencing [33], we reasoned that this apoptotic component may be more pronounced in HeLa cells than in, for example, SiHa cells. In support of this hypothesis, the apoptosis markers cleaved PARP (Poly(ADP‐ribose)‐Polymerase) and cleaved Caspase 9 were found to be substantially more induced upon combined silencing of B‐MYB and A‐MYB in HeLa, compared to SiHa cells (Supporting Information S1: Figure S4).

Taken together, these findings show that the pro‐senescent response of normoxic cells towards B‐MYB silencing can be substantially increased by concomitant A‐MYB silencing. This observation indicates that the induction of A‐MYB can, at least partially, counteract the pro‐senescent effects of B‐MYB silencing in normoxic cells and that combined repression of B‐MYB and A‐MYB is required for highly efficient induction of cellular senescence under normoxia.

### 3.5 Hypoxic Cells Lack the Compensatory Induction of A‐MYB In Response to B‐MYB Silencing and Are Particularly Sensitive to the Pro‐Senescent Effects of B‐MYB Repression

**Figure 5:**
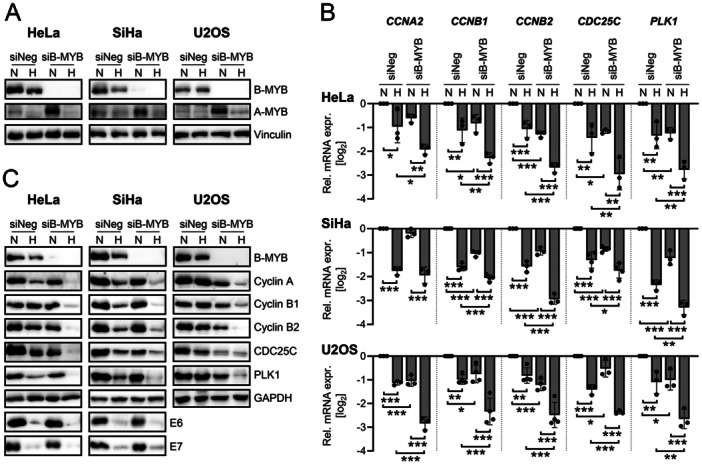
Hypoxic cells lack the compensatory induction of A‐MYB in response to B‐MYB silencing. HeLa, SiHa, or U2OS cells were transfected with siB‐MYB, or control siRNA (siNeg). 24 h post‐transfection, cells were cultivated under normoxia or hypoxia and subsequently harvested after 48 h for RNA and protein analyses, with the exception of SiHa cells. SiHa cells were harvested after 24 h for RNA analyses. (A) Immunoblot analyses of B‐MYB, A‐MYB, and Vinculin protein levels. (B) qRT‐PCR analyses of *CCNA2*, *CCNB1*, *CCNB2*, *CDC25C*, and *PLK1* mRNA levels. Individual data points and mean expression levels with SD (*n* ≥ 3), relative to the expression of siNeg at normoxia are shown (log_2_). Statistical significance was evaluated using two‐way ANOVA. *p ≤ 0.05, **p ≤ 0.01, and ***p ≤ 0.001. (C) Immunoblot analyses of B‐MYB, Cyclin A, Cyclin B1, Cyclin B2, CDC25C, PLK1, E6, E7, and GAPDH protein levels.

In view of this critical interplay between B‐MYB and A‐MYB in the senescence control of normoxic cells, this regulatory phenomenon was next investigated in hypoxic cells. Strikingly, and in remarkable contrast to the strong induction of A‐MYB levels upon B‐MYB repression in normoxic cells, this response was absent in hypoxic cells (Figure 5A). This finding indicates that hypoxic cells are strongly impaired in their ability to functionally compensate for the loss of B‐MYB through upregulation of A‐MYB levels. If this holds true, it would be expected that B‐MYB silencing results in a more efficient downregulation of B‐MYB target genes under hypoxia (no compensatory A‐MYB induction), compared to normoxia (compensatory A‐MYB induction). In support of this notion, the expression levels of several B‐MYB target genes (Figure 5B) and their protein products (Figure 5C) were substantially more repressed upon B‐MYB silencing in hypoxic cells than in normoxic cells.

**Figure 6:**
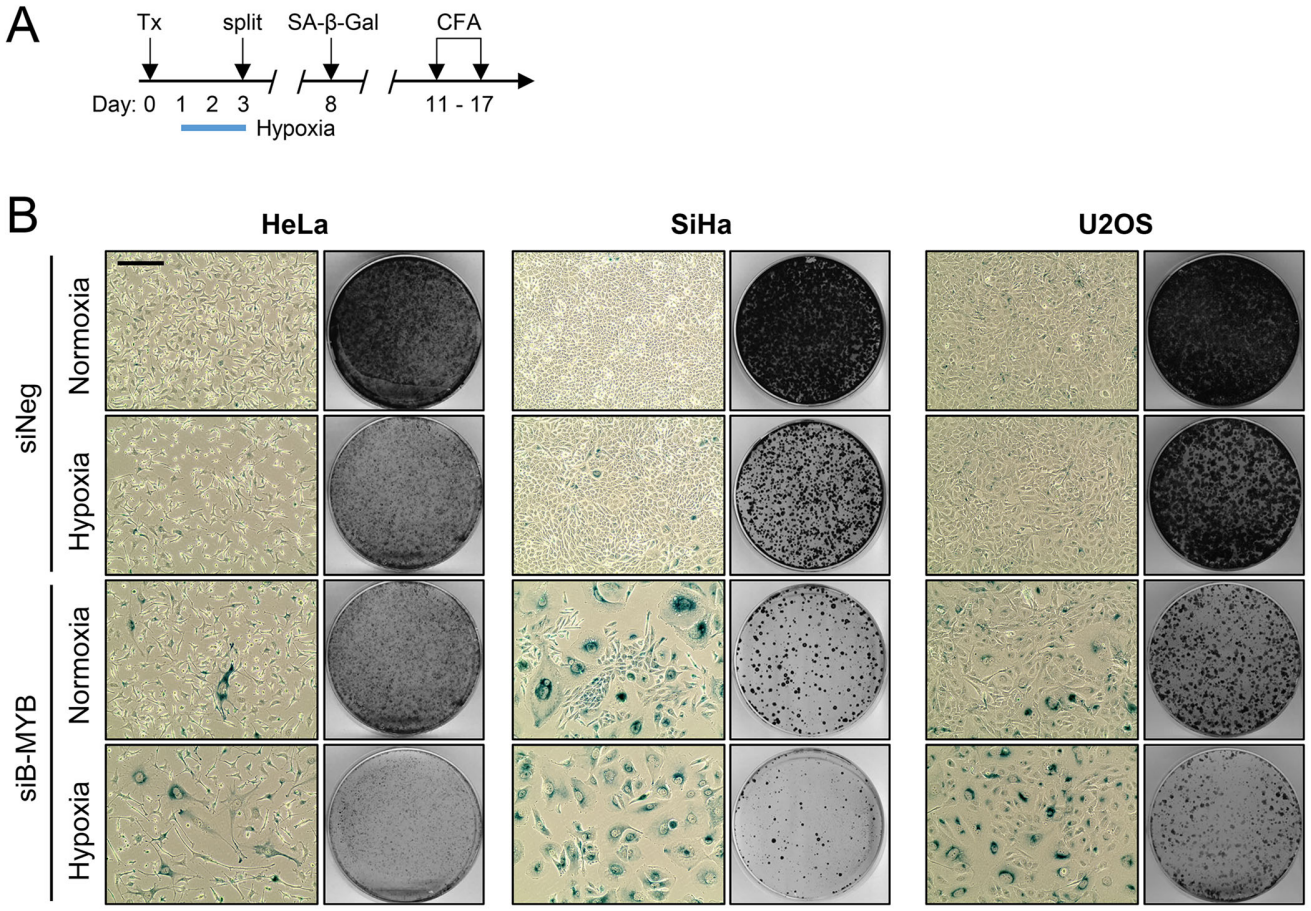
Hypoxic cells are more vulnerable to the pro‐senescent effects of B‐MYB silencing than normoxic cells. (A) Treatment scheme: HeLa, SiHa, or U2OS cells were transfected (Tx) with siB‐MYB, or control siRNA (siNeg). 24 h post‐transfection cells were cultivated under normoxia or hypoxia for 48 h. Cells were split, and fixed after the indicated time periods for senescence assays (SA‐β‐Gal staining) or CFAs. (B) SA‐β‐Gal assays (left panels; positive cells staining blue) (scale bar: 200 µm) and corresponding CFAs (right panels) of HeLa, SiHa, or U2OS cells.

Moreover, since we found that silencing of A‐MYB expression increased the pro‐senescent effect of B‐MYB repression in normoxic cells (Figure 4B and Supporting Information S1: Figure S3D), we hypothesized that hypoxic cells could be particularly sensitive to the downregulation of B‐MYB, due to their lack of compensatory A‐MYB induction. We therefore comparatively analyzed the senescence response of normoxic and hypoxic cells, following the treatment scheme depicted in Figure 6A. Notably, compared to the response of normoxic cells, we found that B‐MYB repression in hypoxic cells led to a strong reduction of cells escaping senescence (i.e. SA‐β‐Gal‐negative cells, lacking morphological signs of senescence) and to an increase of SA‐β‐Gal‐positive hypoxic HeLa, SiHa, and U2OS cells (Figure 6B, left panels for each cell line). In support of this increased pro‐senescent response of hypoxic cells, the inhibitory effects on their colony formation capacity were more pronounced upon silencing B‐MYB under hypoxia, compared to normoxia (Figure 6B, right panels for each cell line).

Collectively, these findings indicate that hypoxic cells are particularly vulnerable to the pro‐senescent effect of B‐MYB inhibition, since they can not functionally compensate for the loss of B‐MYB activity through the upregulation of A‐MYB.

## 4 Discussion

The results of this study provide novel insights into the senescence control of HPV‐positive cancer cells, both under normoxia and hypoxia. As major findings, we firstly uncover that the interconnection between B‐MYB and its paralog A‐MYB is a critical determinant for the senescence response of HPV‐positive cancer cells. In specific, we demonstrate that the compensatory induction of A‐MYB expression counteracts the pro‐senescent effects of B‐MYB loss in normoxic cells. Thus, efficient induction of senescence under normoxia requires the combined inhibition of both B‐MYB and A‐MYB. Secondly, whereas B‐MYB repression also acts pro‐senescent in hypoxic cells, we found that these cells are strongly impaired in their ability to compensate for a B‐MYB loss due to the lack of A‐MYB induction. Further, we provide evidence that these regulatory phenomena are not limited to HPV‐positive cancer cells, but also relevant in HPV‐negative tumor cells. Collectively, our findings indicate that hypoxic cancer cells are particularly vulnerable to the pro‐senescent effects of B‐MYB inhibition, since they lack a functionally compensatory A‐MYB induction.

By analyzing the senescence response of normoxic HPV‐positive cancer cells, we observed that silencing of HPV *E6/E7* oncogene expression, which results in a rapid and efficient induction of senescence [6, 7, 15, 28], was linked to a strong downregulation of B‐MYB levels. In stark contrast, in hypoxic cells–which also downregulate HPV E6/E7 expression, but do not senesce [15]–we found that B‐MYB levels were largely maintained. Similarly, B‐MYB levels were also maintained in hypoxic HPV‐negative U2OS cells. In line with the broad anti‐senescent potential of B‐MYB [19, 20], including in HeLa cells [36], we found that B‐MYB repression induces senescence in all investigated cell lines under normoxia, independent of their HPV status. Interestingly, however, although RNAi‐mediated B‐MYB silencing was highly efficient, senescence induction was only partial. These observations raised the question about the mechanism that restricts the pro‐senescent response towards B‐MYB inhibition.

A recent study has shown that normoxic cells can functionally compensate for B‐MYB loss by increasing A‐MYB levels [33]. Thus, the pro‐senescent effect of B‐MYB silencing may be dampened by the compensatory induction of A‐MYB. Indeed, whereas we found that B‐MYB silencing under normoxia led to a substantial increase of A‐MYB levels in all investigated cell lines, their pro‐senescent response to B‐MYB silencing was substantially increased by concomitant A‐MYB silencing. Further, compared to B‐MYB silencing alone, the combined downregulation of B‐MYB and A‐MYB led to strongly enhanced repression of B‐MYB targets involved in G2/M regulation, correlating with a strongly increased G2/M arrest in accompanying cell cycle analyses. These repressed factors consistently included PLK1 and CDC25C, whose downregulation has been linked to senescence induction in various experimental settings [37, 38, 39, 40, 41, 42]. Collectively, these findings reveal that the compensatory induction of A‐MYB plays an important role for restricting the pro‐senescent effects of B‐MYB inhibition in normoxic cells.

**Figure 7:**
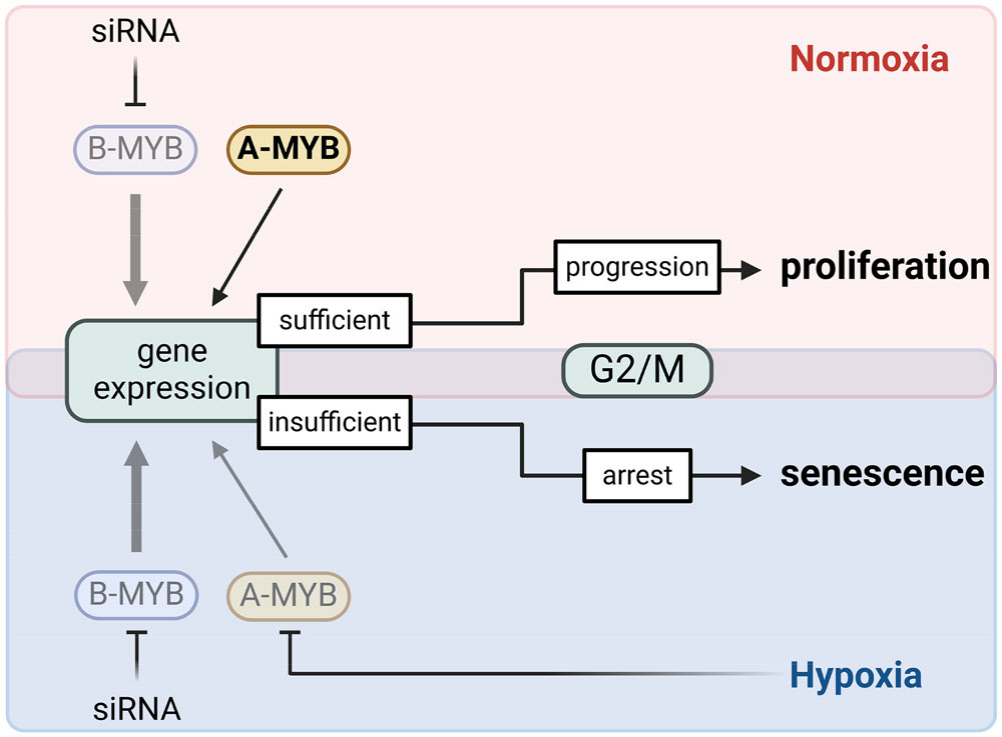
Model for the increased susceptibility of hypoxic cells to the pro‐senescent effects of B‐MYB silencing. In normoxic cells (upper part of the figure), B‐MYB silencing leads to increased expression levels of its paralog, A‐MYB. In turn, A‐MYB can, at least partially, functionally compensate for the loss of B‐MYB by ensuring sufficient expression of cell cycle‐promoting genes critical for G2/M progression, thereby facilitating cell proliferation. In contrast, hypoxic cells (lower part of the figure) are strongly impaired in their ability to increase A‐MYB levels in response to B‐MYB loss. This is linked to insufficient expression of key cell cycle‐promoting genes, inhibition of cell cycle progression, and increased cellular senescence. Created in BioRender. https://BioRender.com/y7z0hy3.

Interestingly, and in striking contrast to normoxic cells, we found that hypoxic cells are strongly impaired in their ability to increase A‐MYB levels in response to B‐MYB loss. This indicates that hypoxic cells are not able to functionally compensate for the loss of B‐MYB. This notion is supported by analyses of B‐MYB target genes, which we found to be substantially more downregulated by B‐MYB silencing in hypoxic cells than in normoxic cells. Based on our data on the role of B‐MYB and A‐MYB for the senescence control of normoxic cells, we further hypothesized that hypoxic cells are more vulnerable to B‐MYB inhibition. Indeed, we found that the senescence response of hypoxic cells is more efficient upon B‐MYB silencing in hypoxic cells, compared to normoxic cells. A tentative model for the increased susceptibility of hypoxic cells towards the pro‐senescent effect of B‐MYB silencing is illustrated in Figure 7.

Although we primarily focused on the analysis of HPV‐positive cervical cancer cells, it is important to note that the interconnection between B‐MYB and A‐MYB is also relevant for the senescence control in the two HPV‐negative cancer cell lines (U2OS, HCT116) tested in our study. This applies to both the enhancement of the pro‐senescent effect of B‐MYB repression through concomitant A‐MYB repression in normoxic cells as well as for the lack of compensatory A‐MYB induction and the increased pro‐senescent sensitivity of hypoxic cells to B‐MYB silencing. These findings show that both of these regulatory principles can occur in an HPV‐independent manner. It will be interesting to further evaluate the conservation of this regulatory mechanism between different cancer types, which requires a more comprehensive analysis of tumor cells derived from different histopathological backgrounds.

Based on the growth‐promoting activities of B‐MYB in cancer cells as well as on its overexpression and linkage to poor prognosis in multiple malignancies (including cervical cancer, colon cancer, breast cancer, ovarian cancer, bladder cancer, non‐small cell lung cancer, and hepatocellular carcinoma) [34, 43, 44, 45, 46, 47, 48], there are intense efforts to generate specific B‐MYB inhibitors and to assess their potential for cancer therapy [20, 49, 50]. Our findings show that the efficacy of senescence induction by targeted B‐MYB inhibition is counteracted by A‐MYB in normoxic cancer cells, but can be increased by concomitant A‐MYB inhibition. Further, a recent study [33] showed that the pro‐apoptotic component of B‐MYB silencing can be enhanced by concomitant A‐MYB silencing. Taken together, these findings indicate that therapeutic strategies aiming at the induction of senescence and/or apoptosis in cancer cells through B‐MYB inhibitors could benefit from additional A‐MYB repression.

Moreover, our findings regarding the senescence regulation in hypoxic cells could also have implications for the clinic. In specific, hypoxic cancer cells typically show increased resistance to various pro‐senescent and pro‐apoptotic stimuli, which could contribute to their increased therapy resistance [15, 51, 52, 53, 54]. Therefore, therapeutic strategies to target hypoxic cancer cells are of high interest and several possible approaches are currently under intense investigation, including the application of metabolic modulators, hypoxia‐activated prodrugs or hypoxia‐targeting bacteria [54, 55]. Our finding that hypoxic cells are particularly sensitive to B‐MYB silencing raises the interesting possibility that B‐MYB inhibitors could not only be useful for repressing the growth of oxygenated cancer cells, but may have the additional benefit to target the therapeutically challenging population of hypoxic cancer cells.

## Author Contributions

All authors contributed to the study conception and design. Material preparation, data collection and analysis were performed by Milica Velimirović, Alicia Avenhaus, Claudia Lohrey, Julia Bulkescher, Felix Hoppe‐Seyler and Karin Hoppe‐Seyler. The first draft of the manuscript was written by Milica Velimirović, Karin Hoppe‐Seyler, and Felix Hoppe‐Seyler and all authors commented on previous versions of the manuscript. All authors have read and agreed to the published version of the manuscript.

## Conflicts of Interest

The authors declare no conflicts of interest.

## Supporting information

Supporting Information Revised.

## Data Availability

The data that support the findings of this study are available from the corresponding author upon reasonable request.
