# Supplementary material for: Hypoxic HPV‐Positive Cancer Cells Are Particularly Sensitive to the Pro‐Senescent Effects of B‐MYB Repression Due to the Lack of Compensatory A‐MYB Induction
Source: J Med Virol. 2025 May 30;97(6):e70422. doi: 10.1002/jmv.70422 (PMC12123558; doi:10.1002/jmv.70422)
Supplement: Supplementary file 1 — Supporting Information Revised. [file JMV-97-e70422-s001.pdf]

Supporting Information

Figure S1

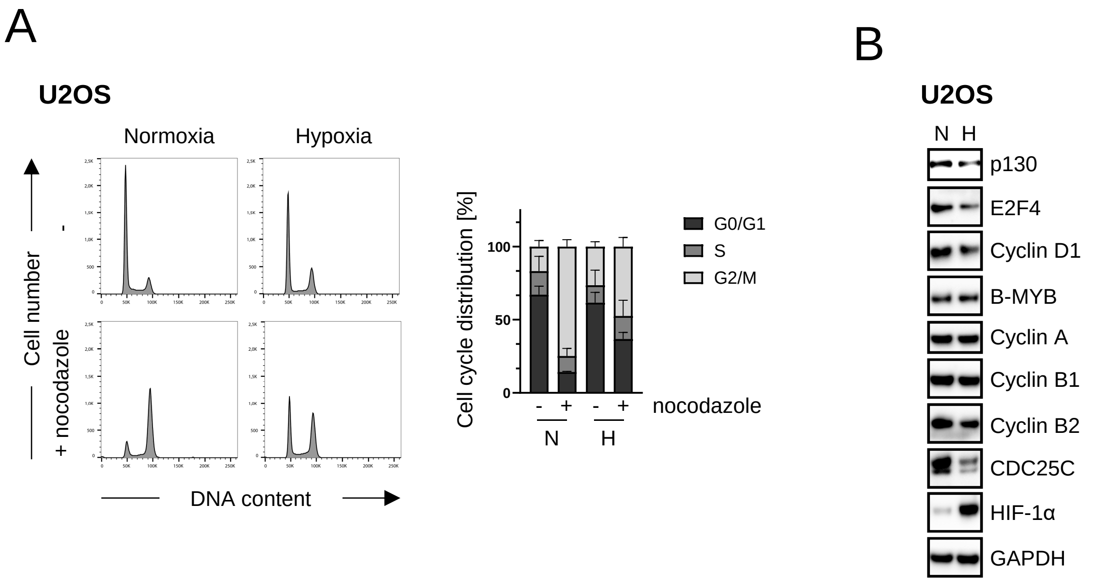

Figure S1. The effects of hypoxia on cell cycle distribution and expression of cell cycle regulators in U2OS cells. (A) U2OS cells were cultivated under normoxia (N; 21% O_2) or hypoxia (H; 1% O_2) for 48 h. Nocodazole or solvent control were added for the last 24 h of cultivation, when indicated. Cells were analyzed by flow cytometry and cell cycle profiles from representative experiments are shown (left panels). Averaged percentages of cell populations in the individual cell cycle phases from 2 different experiments with SD are indicated (right panels). (B) U2OS cells were cultivated under normoxia or hypoxia for 24 h. Immunoblot analyses of p130, E2F4, Cyclin D1, B-MYB, Cyclin A, Cyclin B1, Cyclin B2, CDC25C, hypoxia marker HIF-1α, and GAPDH protein levels.

Figure S2

A

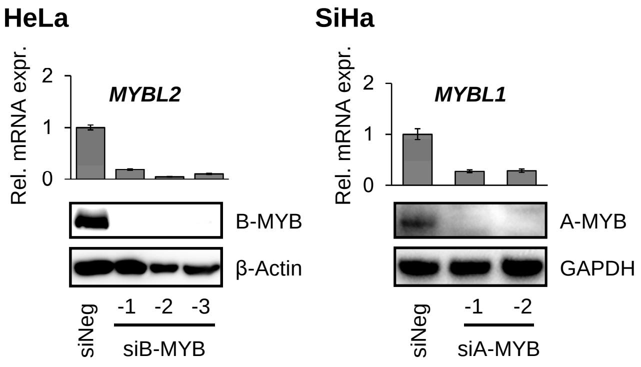

B

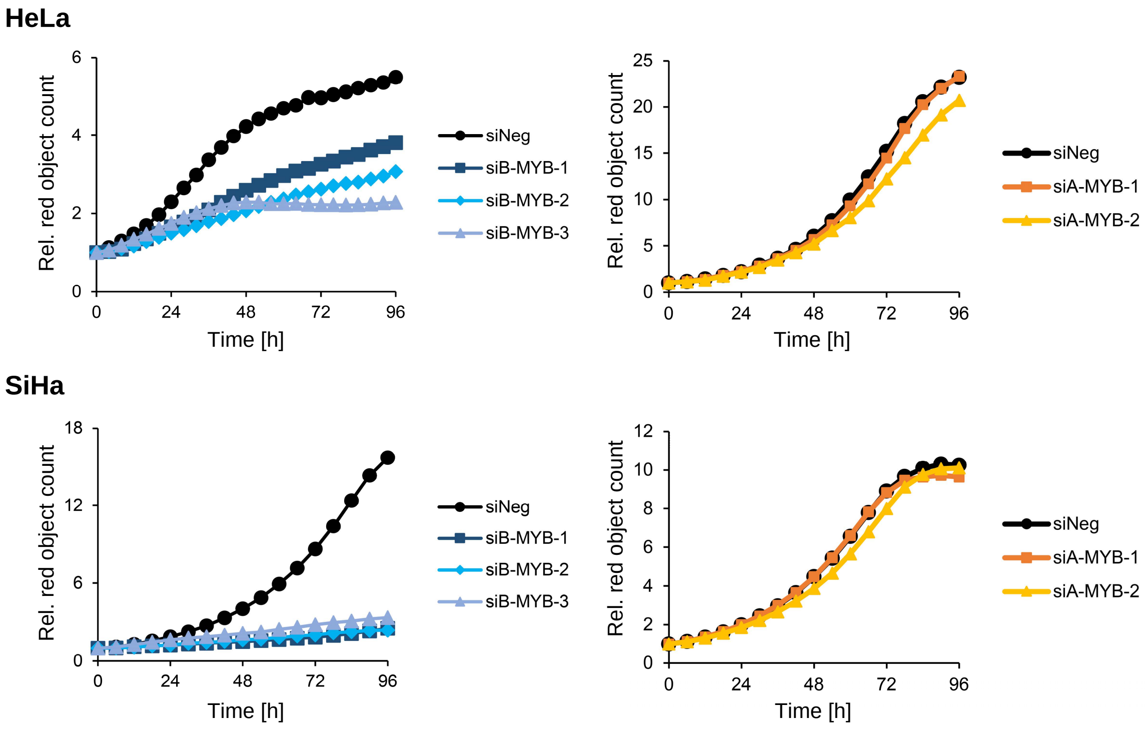

Figure S2. Validation of the employed siRNAs. (A) Three different B-MYB-specific siRNAs (siB-MYB-1, -2, -3) and two different A-MYB-specific siRNAs (siA-MYB-1, -2) were transfected individually into HeLa or SiHa cells. Cells were harvested after 72 h for RNA or protein analyses. qRT-PCR analyses of *MYBL2* (coding for B-MYB) and *MYBL1* (coding for A-MYB) mRNA levels (upper panels) and immunoblot analyses of B-MYB, A-MYB, β-Actin, and GAPDH protein levels (lower panels) are shown. All siRNAs efficiently suppress target gene expression at the transcript and protein level. (B) Live-cell imaging (Incucyte® S3 live-cell imaging system) was used to determine proliferation of HeLa-mKate2 or SiHa-mKate2 cells transfected with siB-MYB-1, -2, -3, or siA-MYB-1, -2, or control siRNA (siNeg) and cultivated under normoxia for up to 96 h. For all further experiments, equimolar pools of the respective siRNAs were used (referred to in the text as siB-MYB or siA-MYB).

Figure S3

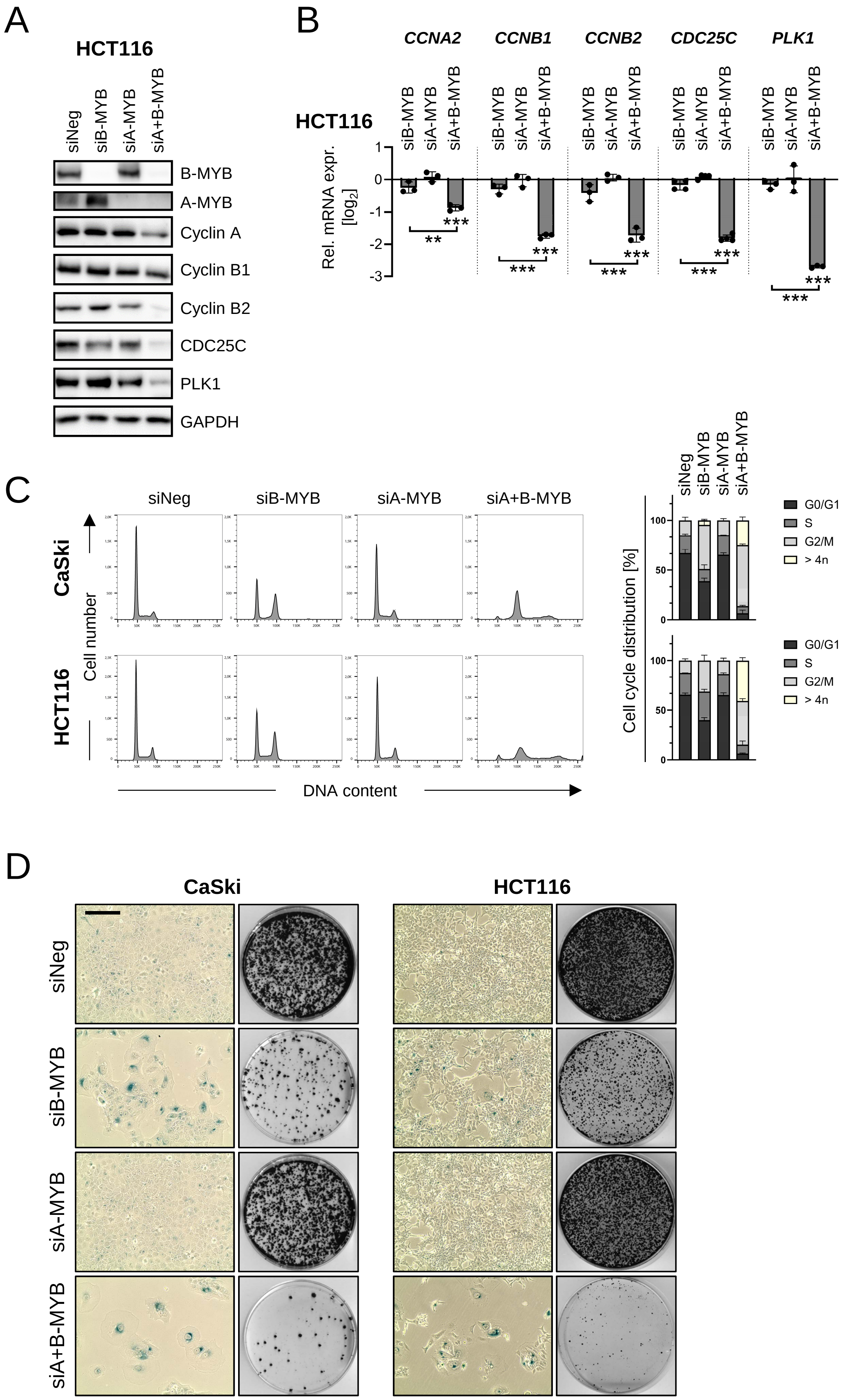

Figure S3. *See figure legend on the next page.*

Figure S3

Figure S3. A-MYB knockdown enhances effects of B-MYB silencing on cell cycle regulation and senescence induction in CaSki and HCT116 cells. CaSki or HCT116 cells were transfected with siA-MYB, siB-MYB, either alone or in combination, or control siRNA (siNeg). Cells were harvested after 72 h for RNA or protein analyses. Cells were split, and fixed following the treatment scheme depicted in Figure 4A for senescence assays (SA-β-Gal staining) or for CFAs. (A) Immunoblot analyses of B-MYB, A-MYB, Cyclin A, Cyclin B1, Cyclin B2, CDC25C, PLK1, and GAPDH protein levels in HCT116 cells. (B) Corresponding qRT-PCR analyses of *CCNA2*, *CCNB1*, *CCNB2*, *CDC25C*, and *PLK1* mRNA levels. Individual data points and mean expression levels with SD (n=3), relative to the expression of siNeg are shown (log_2). Statistical significance was evaluated using one-way ANOVA. * $p \leq 0.05$, ** $p \leq 0.01$, and *** $p \leq 0.001$. (C) Cells were analyzed by flow cytometry and cell cycle profiles from representative experiments are shown (left panels). Averaged percentages of cell populations in the individual cell cycle phases from 3 different experiments with SD are indicated (right panels). (D) SA-β-Gal assays (left panels; positive cells staining blue) (scale bar: 200 µm) and corresponding CFAs (right panels) of CaSki or U2OS cells.

Figure S4

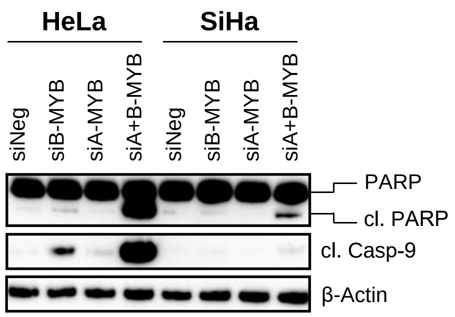

Figure S4. Effects of A-MYB repression on the pro-apoptotic response of HeLa and SiHa cells towards B-MYB silencing. HeLa or SiHa cells were transfected with siA-MYB, siB-MYB, either alone or in combination, or control siRNA (siNeg) and harvested after 48 h (HeLa) or 72 h (SiHa). Immunoblot analyses of apoptosis markers cleaved PARP (cl. PARP) and cleaved Caspase 9 (cl. Casp-9), and β-Actin protein levels.
